# Risk Factors and Clinical Impact of Extended-Spectrum Beta-Lactamase (ESBL)-Producing *Escherichia coli* Bacteremia Among Hospitalized Patients

**DOI:** 10.3390/antibiotics14090882

**Published:** 2025-09-01

**Authors:** Tri Pudy Asmarawati, Fikri Sasongko Widyatama, Hari Basuki Notobroto, Nasronudin Nasronudin, Motoyuki Sugai, Kuntaman Kuntaman

**Affiliations:** 1 Doctoral Programme of Medical Science, Faculty of Medicine, Universitas Airlangga, Surabaya 60131, Indonesia; tpasmarawati@fk.unair.ac.id; 2 Department of Internal Medicine, Faculty of Medicine, Universitas Airlangga, Surabaya 60131, Indonesia; 3 Department of Internal Medicine, Universitas Airlangga Hospital, Surabaya 60115, Indonesia; 4 Department of Medical Microbiology and Parasitology, Faculty of Medicine, Universitas Airlangga, Surabaya 60131, Indonesia; fikri.sasongko@fk.unair.ac.id; 5 Division of Biostatistics and Population Studies, Faculty of Public Health, Universitas Airlangga, Surabaya 60115, Indonesia; haribasuki.n@fkm.unair.ac.id; 6 Institute Tropical of Diseases, Universitas Airlangga, Surabaya 60115, Indonesia; 7 AMR Research Center, National Institute of Infectious Diseases, Japan Institute for Health Security, Tokyo 102-0071, Japan; sugai@niid.go.jp; 8 Department of Antimicrobial Resistance, Hiroshima University Graduate School of Biomedical Science, Hiroshima 739-0046, Japan; 9 Department of Clinical Microbiology, Dr. Soetomo General Academic Hospital, Surabaya 60286, Indonesia

**Keywords:** bacteremia, ESBL-producing *Escherichia coli*, risk factors, infectious diseases, antimicrobial resistance

## Abstract

**Background/Objectives**: The prevalence of ESBL-producing *Escherichia coli* (*E. coli*) has increased significantly, impacting prognoses due to delayed or limited treatment options. We aimed to determine the demographic patterns, risk factors, and clinical outcomes of ESBL-producing *E. coli* in a top-referral hospital in Indonesia. **Methods**: This study was observational in design and focused on hospitalized patients with bacteremia due to *E. coli* during 2022–2024. **Results**: We identified 224 patients during the study period. The median of length of stay was 7 (3–13) days. Mortality occurred in 149 (66.55%) patients, and there was no difference in the mortality between patients with ESBL *E. coli* and those with non-ESBL *E. coli*. The severity of illness, as defined by the Pitt bacteremia score (PBS), was higher in the ESBL *E. coli* group. Urinary tract infection (UTI), previous antibiotic use, and central venous catheter (CVC) insertion were independent risk factors for bacteremia due to ESBL *E. coli* bacteremia. Male gender, shorter length of stay (LOS), solid tumor, pneumonia, mechanical ventilation, CVC insertion, inappropriate initial antibiotic therapy (IIAT), and sequential organ failure assessment (SOFA) score were risk factors for mortality in bacteremia caused by *E. coli*, including both ESBL and non-ESBL producers. Male gender, shorter LOS, CVC usage, and SOFA score were independent risk factors for mortality in bacteremia due to ESBL *E. coli*. **Conclusions**: ESBL-producing *E. coli* increases the severity of bacteremia. Recognizing patients at high risk for ESBL-producing *E. coli* infections is crucial for initiating appropriate empirical antibiotic treatment targeting ESBL-producing pathogens.

## 1. Introduction

The spread of ESBL-producing *Enterobacterales* has increased worldwide in recent years [1,2]. Patients with bacteremia due to ESBL-producing *Enterobacterales* have a poor prognosis due to delayed or limited options for appropriate antibiotic therapy [3].

The results of the antimicrobial resistance (AMR) surveillance work carried out by the World Health Organization (WHO) in 2022 demonstrated that the proportion of *E. coli* from bloodstream infections resistant to cefotaxime and ceftriaxone in Indonesia was 69.7% [4]. The prevalence of *E. coli* resistant to third-generation cephalosporins has remained high for the past 5 years at 69.7–75.6% [5]. The most recent systematic review and meta-analysis reports that the prevalence of ESBL-producing *E. coli* in Indonesia is 57.84% (95% CI: 45.97–69.72%) [6]. Meanwhile, the most consumed antibiotics in Indonesia were beta-lactams, especially cephalosporins and penicillin group [7].

Infections caused by multidrug-resistant bacteria, including ESBL *E. coli*, increase mortality risk [8,9,10]. Predictors of bacteremia mortality due to ESBL *E. coli* are important to explore in the clinical management of patients to improve the prevention and treatment strategies for better outcomes [11]. However, studies regarding the clinical aspects of bacteremia caused by ESBL *E. coli* are still limited and reveal mixed results [3,8,12]. Previously published research recommends risk factors for ESBL that are specific to each healthcare institution [13]. The elucidation of risk factors for developing ESBL *E. coli* bacteremia and mortality related to ESBL *E. coli* bacteremia is crucial [14]. It may also be important to guide effective empirical antibiotic therapy [1]. Based on these challenges and the increasing prevalence of ESBL *E. coli* in Indonesia, we conducted a study to determine the clinical impact and epidemiological pattern of risk factors contributing to bacteremia due to ESBL-producing *E. coli.*

## 2. Results

### 2.1. Baseline Characteristics

We observed 224 patients with *E. coli* bacteremia during the study period. The prevalence of ESBL *E. coli* was 61.6% (138 out of 224). Demographic characteristics suggested that sex and age did not lead to differences between the ESBL *E. coli* group and the non-ESBL *E. coli* group (Table 1). The median length of hospital stay was 7 (3–13) days. The most common comorbidity was diabetes mellitus with hypertension, followed by solid tumors. Comorbidity scoring using the Charlson comorbidity index (CCI) showed no significant difference between the two groups (*p* = 0.552).

Common potential sources of bacteremia are pneumonia, urinary tract infections, and intra-abdominal infections. As many as 179 out of 224 patients (81%) used urinary catheters. Healthcare-associated infection occurred in 54 (24.1%) patients. As many as 52.2% of the subjects received cephalosporin as an initial empirical antibiotic. Mortality occurred in 149 (66.55%) of all patients, and there was no difference in mortality between the two groups.

**Table 1:** Characteristics of patients with bacteremia caused by *E. coli.*

Variables	*Escherichia coli*		
	ESBL (*n* = 138)	Non-ESBL (*n* = 86)	*p*
Demographics			
Gender, male	60 (43.5)	33 (38.4)	0.451
Median age, years (IQR)	53.5 (42.75–63)	54 (40–64)	0.584
Median LOS (IQR)	8 (3–15.25)	6 (3–11)	0.147
Comorbidities			
Hypertension	49 (35.5)	29 (33.7)	0.785
Diabetes mellitus	54 (39.1)	27 (31.4)	0.241
Heart failure	12 (8.7)	9 (10.5)	0.659
COPD	3 (2.2)	2 (2.3)	0.940
Liver cirrhosis	7 (5.1)	6 (7.0)	0.553
Hematologic malignancy	8 (5.8)	7 (8.1)	0.495
Solid tumor	32 (23.2)	22 (25.6)	0.684
HIV/AIDS	0 (0)	1 (1.2)	0.204
Median CCI (IQR)	4 (1–6)	3 (1–5.25)	0.267
CCI ≥ 3	81 (58.7)	47 (54.7)	0.552
Potential source of bacteremia			
Pneumonia	93 (67.4)	60 (69.8)	0.710
Intra-abdominal	32 (23.2)	60 (69.8)	<0.001 *
Urinary tract	93 (67.4)	16 (18.6)	<0.001 *
Intracranial	4 (2.9)	60 (69.8)	<0.001 *
Skin and soft tissue	34 (24.6)	0 (0)	<0.001 *
Primary bloodstream infection	5 (3.6)	2 (2.3)	0.587
Hospital-acquired infection	31 (22.5)	23 (26.7)	0.466
Previous exposure			
Prior hospitalization	102 (73.9)	54 (62.8)	0.078
Prior ICU stay	22 (15.9)	12 (14.0)	0.687
Prior surgery	55 (39.9)	23 (26.7)	0.045 *
Prior chemotherapy or radiotherapy	9 (6.5)	12 (14.0)	0.063
Prior corticosteroid use	15 (10.9)	16 (18.6)	0.103
Prior antibiotic use	76 (55.1)	23 (26.7)	<0.001 *
History of hemodialysis	12 (8.7)	3 (3.5)	0.129
Use of invasive procedures or devices			
Mechanical ventilation	55 (39.9)	29 (33.7)	0.356
Central venous catheterization	86 (62.3)	37 (43.0)	0.005 *
Urinary catheterization	119 (86.2)	62 (72.1)	0.009 *
Laboratory examination			
Leukocytosis	105 (76.1)	53 (61.6)	0.021 *
Neutropenia	6 (4.3)	9 (10.5)	0.075
Serum albumin < 30 g/L	115 (84.6)	63 (74.1)	0.056
Severity of illness			
Median qSOFA score (IQR)	2 (1–3)	1 (1–2)	0.036 *
Median SOFA score (IQR)	6 (4–8)	6 (3–8)	0.576
Median PBS (IQR)	2 (0–4)	0.5 (0–4)	0.001 *
Vasopressor use	67 (48.6)	30 (34.9)	0.045 *
Median CRP (IQR)	17.9 (10.32–28.22)	14.62 (5.31–25.53)	0.091
Median procalcitonin (IQR)	22.75 (3.15–62.99)	24.03 (2.86–50.32)	0.549
Empirical antibiotic treatment	*(n* = 135)	(*n* = 80)	0.136
Cephalosporin	80 (59.3)	37 (46.3)	
Fluoroquinolone	35 (25.9)	31 (38.8)	
BLBLI	16 (11.9)	12 (15)	
Aminoglycosides	2 (1.5)	0 (0)	
Metronidazole	2 (1.5)	0 (0)	
Inappropriate initial antibiotic therapy	115 (83.3)	16 (20.5)	<0.001 *
Mortality, n (%)	97 (70.3)	52 (60.5)	0.130

Note: * = significant at <0.05. Data are expressed as n (%) unless otherwise stated. ESBL: extended-spectrum beta-lactamase; IQR: interquartile range; LOS: length of stay; COPD: chronic obstructive pulmonary disease; HIV/AIDS: human immunodeficiency virus/acquired immunodeficiency syndrome; CCI: Charlson comorbidity index; ICU: intensive care unit; qSOFA: quick sequential organ failure assessment; SOFA: sequential organ failure assessment; PBS: Pitt bacteremia score; CRP: C-reactive protein; BLBLI: beta-lactam–beta-lactamase inhibitor.

### 2.2. Analysis of the Development of ESBL-Producing E. coli Bacteremia

Bivariate analysis between the ESBL *E. coli* bacteremia group and non-ESBL *E. coli* bacteremia is shown in Table 1. Age, gender, and hospital length of stay (LOS) showed no differences between the two groups. The comorbidity condition and CCI score also did not show any differences. Urinary tract infections, along with a history of surgery and antibiotic use, were more common in patients with ESBL-producing *E. coli* bacteremia. The use of invasive medical devices such as CVCs and urinary catheters was also higher in patients with bacteremia due to ESBL *E. coli*. Median CRP and procalcitonin levels showed no significant difference between the groups, while the median PBS was higher in the ESBL *E. coli* group. The ESBL *E. coli* group more often received IIAT (83.3%).

The logistic regression model was significant (X^2^ = 202.66, *p* < 0.001 (Omnibus Test)), explaining 81% of the variance (Nagelkerke R^2^ = 0.81) and correctly classifying 91.1% of cases. The Hosmer–Lemeshow goodness-of-fit test was not significant (X^2^ = 5.76, *p* = 0.67), indicating that the model fit the data well. The logistic regression model had an event per variable (EPV) of 23, which meant the model was considered to have stable estimates. Urinary tract infection (*p* = 0.002; OR = 5.876), previous antibiotic use (*p* = 0.011; OR = 4.563), and CVC insertion (*p <* 0.001; OR = 10.590) were independent risk factors for ESBL *E. coli* bacteremia (Table 2). Intra-abdominal infection and intracranial infection showed an inverse association with bacteremia due to ESBL *E. coli* in this study.

### 2.3. Analysis of Mortality Among E. coli Bacteremia Patients

The results of the bivariate analysis of mortality-related variables in patients with bacteremia caused by *E. coli* are presented in Table 3. Male gender, the presence of pneumonia, and intra-abdominal infections were associated with higher mortality rates. Patients with mortality had a shorter period of hospitalization compared to the survivor group (5 vs. 11 days). Patients who experienced mortality were also more likely to have received treatment with invasive medical equipment, such as mechanical ventilators, urinary catheters, and CVCs. ESBL-producing *E. coli* had a similar outcome to non-ESBL *E. coli* bacteremia. Patients with hypoalbuminemia had higher mortality than those with normal albumin levels. Mortality was also higher among patients who received IIAT (*p* = 0.036). The parameters of disease severity, including qSOFA score, SOFA score, PBS, and vasopressor use, showed significant differences between the two groups. In contrast, CRP and procalcitonin showed no significant differences.

The logistic regression model was significant (X^2^ = 105.77, *p* < 0.001 (Omnibus Test)), explaining 55% of the variance (Nagelkerke R^2^ = 0.55) and correctly classifying 81.7% of cases. The Hosmer–Lemeshow goodness-of-fit test was not significant (X^2^ = 4.92, *p* = 0.77), indicating that the model fit the data well. The model achieved an EPV ratio of 10.64, exceeding the recommended threshold of 10 for logistic regression. The multivariate analysis revealed that the risk factors for mortality in bacteremia due to *E. coli* included male gender (*p* = 0.003; OR = 3.646), shorter LOS (*p* < 0.001; OR = 0.890), solid tumor (*p* = 0.011; OR = 3.654), pneumonia (*p* = 0.015; OR = 2.826), mechanical ventilation usage (*p* = 0.041; OR = 2.976), CVC usage (*p* = 0.037; OR = 2.498), IIAT (*p* = 0.030; OR = 2.403), and SOFA score (*p* < 0.001; OR = 1.371) (Table 4).

### 2.4. Risk Factors for Mortality Among Patients with ESBL-Producing E. coli Bacteremia

We analyzed several variables related to mortality among 138 patients with bacteremia caused by ESBL *E. coli* (Table 5). Males had a higher mortality due to bacteremia than females (*p* = 0.01). Patients who died also had a shorter median LOS than those who survived (*p* < 0.001). Among observed comorbidities, hypertension was more prevalent in patients who survived than in the non-survivor group. Regarding the source of bacteremia, intra-abdominal infections led to a higher proportion of mortality (27.8% vs. 12.2%). Subjects who required mechanical ventilators, CVCs, and urinary catheters had a significantly higher mortality risk, with *p*-values of 0.002, 0.019, and 0.004, respectively. Serum albumin levels < 30 g/L led to a higher risk of mortality. The severity parameters indicated by the qSOFA score, SOFA score, PBS, and vasopressor use were associated with higher mortality.

The logistic regression model was significant (X^2^ = 67.82, *p* < 0.001 (Omnibus Test)), explaining 56% of the variance (Nagelkerke R^2^ = 0.56) and correctly classifying 83.8% of cases. The Hosmer–Lemeshow goodness-of-fit test was not significant (X^2^ = 10.12, *p* = 0.257), indicating that the model fits the data adequately. The EPV ratio was 8.81, which is slightly below the recommended threshold of 10 for logistic regression. Risk factors associated with mortality due to ESBL *E. coli* bacteremia included male gender (*p* = 0.007; OR 4.927), LOS (*p* = 0.004; OR = 0.917), CVC usage (*p* = 0.004; OR = 4.885), and SOFA score (Table 6). The presence of hypertension had an inverse relationship with the mortality of bacteremia patients due to ESBL *E. coli*.

## 3. Discussion

The prevalence of ESBL in *E. coli* specimens in this study was relatively high—61.6%—compared to other studies [5,15,16]. Demographic patterns showed that subjects of the male gender with bacteremia both due to *E. coli* and ESBL *E. coli* tended to experience higher mortality. A similar study involving 554 patients over 16 years reported that sex did not differ between patients with bacteremia due to ESBL *E. coli* and non-ESBL *E. coli* [3]. Meanwhile, studies involving adult patients with bacteremia due to *Enterobacteriales* showed that the male sex was associated with non-susceptibility to carbapenem and a poor prognosis at hospitalization [17]. Males have a higher odds ratio (1.51 times) for ESBL production in certain bacteria, such as *E. coli*, compared to females, though the overall effect size when predicting ESBL presence is small [18].

Studies investigating the role of comorbid diseases in the emergence of antibiotic resistance or the severity of infection show varied results [19,20]. Common comorbidities, such as diabetes, hypertension, and renal failure, have been significantly associated with higher mortality in ICU patients with bloodstream infections [19]. Chronic diseases exacerbate the severity of infections through various mechanisms, affecting pathophysiology and treatment outcomes [21]. Unlike our study, another study in patients with bacteremia due to *Enterobacteriales* who had hypertensive comorbidities had a worse prognosis during hospitalization, along with other risk factors, such as non-susceptibility to carbapenem [17]. A multicenter study conducted in Indonesia in patients with bloodstream infection due to carbapenem-non-susceptible *Acinetobacter baumanii* also did not show a significant difference in terms of comorbidities [20]. Comorbid diseases generally increase both the risk of acquiring antibiotic-resistant infections and the severity of infection outcomes [22]. However, the specific effects vary depending on the clinical context, pathogen, and treatment factors [23].

Intra-abdominal infection does not often develop into ESBL and even has an inverse relationship with the occurrence of ESBL. Other studies have reported similar results [13]. Several studies indicate that UTIs are a common source of bloodstream infections caused by ESBL-producing *E. coli*, highlighting the clinical significance of these infections progressing from localized urinary infection to systemic spread [24,25]. Previous research aligns with our study, demonstrating that UTIs are frequently caused by ESBL-producing *E. coli* and often progress to bacteremia [13,24,25,26,27]. Urinary catheter use significantly increases the risk of UTIs caused by ESBL *E. coli* due to catheter-associated colonization and infection, contributing to the spread and persistence of resistant strains in healthcare settings [28,29]. Urinary tract infections further affect mortality among *E. coli* bacteremic patients and those with ESBL *E. coli* bacteremia [11,12].

Previous exposure has a significant influence on the development of infections caused by *E. coli* and other multidrug-resistant organisms (MDROs) [30,31]. Our study showed that prior surgery and antibiotic use were associated with ESBL *E. coli* bacteremia, although they did not impact mortality. Moreover, another study found that patients who received more than one antibiotic in the last 90 days had a three-times-greater risk of developing ESBL infection than patients who did not receive antibiotics [13]. Prior surgery and healthcare exposure, such as hospitalization and residence in nursing facilities, also substantially increase the risk of MDRO infections [30]. Another study highlighted that prior abdominal surgery is associated with a higher likelihood of carrying ESBL-producing *Enterobacteriaceae*, particularly when combined with recent antibiotic exposure [32].

The use of invasive devices significantly contributes to the development of MDRO infections, including ESBL *E. coli* [33,34,35]. Increased risk of colonization was found to be associated with a greater need for mechanical ventilation and urinary catheter use in MDRO infection [33]. Prolonged use of mechanical ventilators, urinary catheters, or CVCs was directly associated with increased microbial risk and higher mortality, which was also evident in our study [33]. Urinary catheterization and invasive genitourinary procedures within the previous 12 months were found to be independent risk factors for the emergence of ESBL uropathogenic *E. coli* (UPEC) bloodstream infection (BSI) [36]. Integrated implementation of infection control and prevention significantly reduces the risk of device-related and ESBL infection [37,38].

The Pitt bacteremia score (PBS) is a widely validated clinical tool used to assess the acute severity of illness and predict mortality risk in patients with BSI [39,40]. Our study showed similar results among patients with *E. coli* bacteremia and ESBL *E. coli* bacteremia. It has served as a stratification tool in important multicenter studies involving BSIs caused by Gram-positive bacteria, Gram-negative bacteria, and Candida species [11,41]. Another study involving 388 *E. coli* bacteremia patients showed that a higher SOFA score was an independent risk factor for mortality of bacteremia patients due to *E. coli* [42]. In contrast with our results, in which ESBL *E. coli* was associated with increased severity and septic shock, but not with mortality, previous research reported that ESBL infections were significantly linked to higher odds of mortality, but not with the progression to septic shock [43,44]. However, one study in Indonesia reported a similar result to our study, although the reported correlation was low [45].

We highlighted the significant proportion of patients receiving inappropriate initial antibiotic therapy among the ESBL *E. coli* bacteremia group and its consequences for the increased mortality risk in our study. Inappropriate empirical antibiotic therapy has been consistently linked to higher mortality rates, particularly in serious infections and in resistant strains of *Enterobacteriales* and *Staphylococcus aureus* [46,47]. Previous cephalosporin use is stated to be a risk factor for the occurrence of ESBL *E. coli* bacteremia [36]. Ceftriaxone, levofloxacin, and ampicillin were the most consumed antibiotics in inpatients [7]. During the COVID-19 pandemic, antibiotic consumption increased further, especially for broad-spectrum agents such as ceftriaxone [48]. In contrast, the prevalence of *E. coli* resistant to third-generation cephalosporins has increased significantly over the past 5 years [11]. Empirical antibiotic guidelines in Indonesia are primarily based on national recommendations, but clinical practice is heavily influenced by local resistance patterns and the availability of antibiotics [49,50,51].

In this study, we found a relatively high mortality rate of 66.5% in bacteremia patients due to *E. coli* and 70.3% among the ESBL group. This proportion is higher than that found in other studies [3,13]. One large multicenter study found that IIAT was associated with 46% increased odds of in-hospital death compared to when patients received appropriate therapy, regardless of whether the patients had sepsis or septic shock [52]. Another phenomenon observed in our study was that patients who experienced mortality had a shorter period of hospitalization than the survivors, while other studies report the opposite [11,53]. This phenomenon is likely to occur in tertiary referral hospitals, where patients have experienced symptoms for a prolonged period, have received treatment at a previous health facility, and are only referred when the severity worsens, complex comorbidities arise, or complications occur [54]. Another reason for shorter treatment in non-survivors includes the rapid progression of sepsis or lack of response to antibiotic therapy, leading to earlier death and hence shorter cumulative antibiotic exposure [53].

One limitation of this study is that it is a single-center study, with guidelines and patterns of antibiotic use that may differ from those of other areas. Data on previous exposure history and the use of invasive medical equipment were retrieved retrospectively from medical records, which may contain incomplete information regarding the duration of exposure experienced by subjects. In addition, we did not evaluate the timing of definitive antibiotic administration or duration of treatment after the onset of bacteremia to explain the phenomenon of short hospitalizations and high mortality in subjects. Further research should involve non-referral hospitals to assess the impact of early definitive therapy and explore gender-related prognostic differences in bacteremia outcome.

## 4. Materials and Methods

### 4.1. Study Design and Patient Selection

This observational study employed a cross-sectional design to investigate the risk factors associated with bacteremia caused by ESBL *E. coli* in hospitalized patients in Dr. Soetomo General Academic Hospital, Surabaya, Indonesia. This study was a continuation of the Tricycle Project for ESBL-producing *E. coli* surveillance in bacteremia, pregnant women, chickens, and the environment conducted in Surabaya, Indonesia. The presence of *E. coli* was determined based on the results of blood cultures to examine the growth of *E. coli* between 2022 and 2024. Inclusion criteria were as follows: (1) a patient with positive blood culture of *E. coli* and signs of infection; (2) age more than 18 years. The exclusion criteria included blood cultures revealing multiple bacteria or fungi, as well as incomplete medical records. Data on demography, comorbidities, potential sources of bacteremia, previous exposure, use of invasive medical equipment, and laboratory results were obtained from medical records.

**Figure 1:**
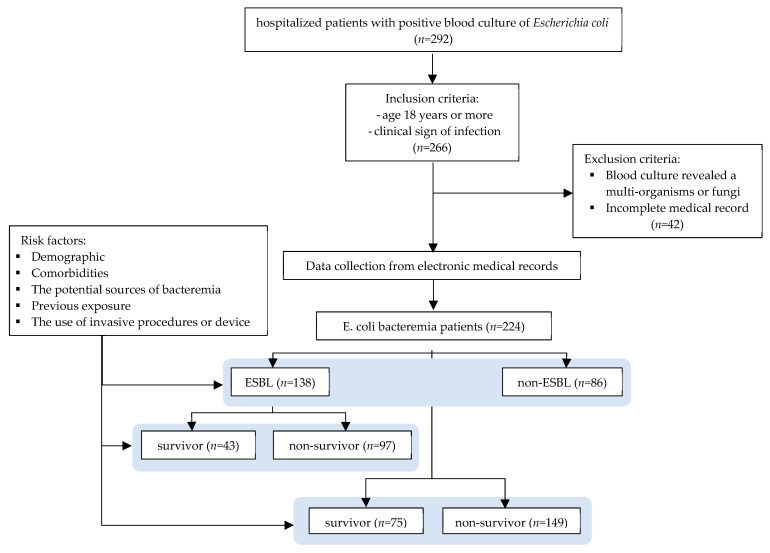
Flowchart of patient selection and data collection.

The comorbidities were calculated using the Charlson comorbidity index (CCI) and included several comorbid conditions, including myocardial infarction, myocardial infarction, peripheral vascular disease, cerebrovascular disease, dementia, chronic pulmonary disease, rheumatic disease, peptic ulcer, hemiplegia, liver disease, chronic kidney disease, leukemia, lymphoma, HIV/AIDS, solid tumors, and diabetes mellitus [55]. Previous exposure variables included previous hospitalization, chemotherapy or radiotherapy, use of corticosteroids (such as prednisone > 20 mg/day or equivalent) for more than 7 days [13], surgery, hemodialysis, use of an antibiotic, and a hospital stay of 30 days before the onset of bacteremia [3]. The use of invasive devices was defined as the insertion of invasive mechanical ventilation, a CVC, or a urinary catheter for more than 48 h before the onset of bacteremia. Laboratory results were recorded at the time of bacteremia onset. Mortality was defined as in-hospital mortality. A flowchart detailing patient selection is provided in Figure 1.

### 4.2. Microbiology Test

The routine protocol used in microbiological testing at the hospital where the research was conducted is as follows. Positive blood culture samples in BD BACTEC^TM^ Plus Aerobic/F culture (cat. 442023, Becton, Dickinson and Company, Sparks, MD, USA) vials were detected using the BD BACTEC^TM^ FX system (Becton, Dickinson and Company, Sparks, MD, USA). Samples with positive growth were subsequently cultured on sheep blood agar and MacConkey agar (Oxoid, Hampshire, UK) and incubated at 37 °C for 18–24 h. The Gram-negative bacterial colonies from the culture were then identified, and the minimum inhibitory concentration of antibiotics was determined using the BD Phoenix^TM^ M50 Automated Microbiology System (Becton, Dickinson and Company, Sparks, MD, USA) instrument with the NMIC/ID 4 panel (cat. 448505, Becton, Dickinson and Company, Sparks, MD, USA). The interpretation of antibiotic resistance was based on the Clinical and Laboratory Standards Institute (CLSI) guidelines 2022 [56]. The ESBL production of *E. coli* was determined based on established algorithms and using the BD Phoenix^TM^ M50 Automated Microbiology System (Becton, Dickinson and Company, Sparks, MD, USA) [57]. The results were used to determine which patients had *E. coli* bacteremia.

### 4.3. Statistical Analysis

#### 4.3.1. Descriptive and Bivariate Analysis

We analyzed the data using SPSS version 24 (Chicago, IL, USA; RRID: SCR_002865). Demographic and characteristic data are presented as the median ± IQR. Data on demographics, comorbidities, potential sources of bacteremia, previous exposure, use of invasive medical equipment, and other categorical data are given as frequency and percentage distributions. Numeric data, such as laboratory results, SOFA score, PBS, C-reactive protein (CRP), and procalcitonin, are presented as median ± IQR.

We analyzed the normality of continuous data using the one-sample Kolmogorov–Smirnov test and obtained a result of *p* < 0.001. Therefore, comparisons between groups, namely the ESBL *E. coli* and non-ESBL *E. coli* groups, survivors and non-survivors in the *E. coli* bacteremia group, and survivors and non-survivors in the ESBL *E. coli* bacteremia group, were carried out using a nonparametric test, the Mann–Whitney test. Categorical variables were examined with the Chi-square test. Significance was set at *p* < 0.05, with a 95% confidence interval.

#### 4.3.2. Multivariate Analysis

Variables with *p* < 0.05 in the bivariate analysis were entered into multiple binary logistic regression models with the forward likelihood ratio method to explore risk factors influencing the development of ESBL *E. coli* bacteremia, mortality related to *E. coli,* and ESBL *E. coli* bacteremia. Logistic regression model adequacy was evaluated by calculating the number of events per variable (EPV), defined as the ratio of outcome events to the number of predictors included in the model, with a threshold of ≥10 EPV considered acceptable. Multicollinearity was assessed using variance inflation factors (VIFs) and the tolerance test. The quick SOFA showed high collinearity (VIF = 6.51), so it was excluded from the analysis. Meanwhile, other variables showing tolerance values of >0.21 and VIF < 4.72 were included in the analysis. Continuous data were analyzed directly via logistic regression. In the analysis of risk factors for bacteremia ESBL *E. coli*, all samples were included in the analysis (*n* = 224). Meanwhile, in the analysis of mortality due to *E. coli* and ESBL *E. coli* bacteremia, missing values were found in the variables CRP (49.6%) and procalcitonin (20.1%), so these two variables were not included in the analysis.

Variables included in the ESBL *E. coli* bacteremia risk factor analysis model included intra-abdominal infections, UTIs, intracranial infection, previous antibiotic use, CVC use, and urinary catheter use. Variables included in the *E. coli* bacteremia mortality analysis model included gender, LOS, solid tumors, pneumonia, intra-abdominal infection, use of mechanical ventilation, CVC and urinary catheter use, leukocytosis, serum albumin < 30 g/L, IIAT, SOFA score, PBS, and vasopressor use. Meanwhile, the variables included in the ESBL *E. coli* bacteremia mortality analysis model included gender, LOS, hypertension, intra-abdominal infection, use of mechanical ventilation, CVC and urinary catheter use, serum albumin < 30 g/L, SOFA score, PBS, and vasopressor use. Model fit was evaluated using the omnibus test of model coefficients, the Hosmer–Lemeshow test, and Nagelkerke R^2^. Results are expressed as odds ratios (ORs) and 95% confidence intervals (CIs), and *p* < 0.05 was considered significant.

## 5. Conclusions

ESBL-producing *E. coli* complicates the severity of bacteremia, although it has no direct effect on fatality rates. Recognizing individuals with risk factors for ESBL-producing *E. coli* infections, specifically urinary tract infections, prior antibiotic usage, and CVC insertion, is crucial for initiating appropriate empirical antibiotic treatment targeting ESBL-producing pathogens. The high overall mortality and shorter hospital stays among non-survivors suggest late-stage referral and delayed treatment. This study highlights the importance of antibiotic stewardship, infection control practices, and patient monitoring for high-risk groups.

## Figures and Tables

**Table 2:** Risk factors of bacteremia caused by ESBL *E. coli.*

Variables	Multivariate Analysis		
	*p*	OR	95% CI for OR
Potential source of bacteremia			
Intra-abdominal	<0.001	0.068	0.021–0219
Urinary tract	0.002	5.876	1.941–17.790
Intracranial	<0.001	0.003	0.001–0.019
Previous exposure			
Prior antibiotic use	0.011	4.563	1.421–14.652
Use of invasive procedures or devices			
Central venous catheterization	<0.001	10.590	3.060–36.657

**Table 3:** Bivariate analysis of mortality caused by *E. coli* bacteremia.

Variables	Bivariate Analysis		
	Survivor (*n* = 75)	Non-Survivor (*n* = 149)	*p*
Demographics			
Male gender	21 (28)	72 (48.3)	0.004 *
Median age, years (IQR)	53 (42–63)	54 (40.5–64)	0.669
Median LOS, days (IQR)	11 (8–18)	5 (2–9.5)	<0.001 *
Time before bacteremia, days (IQR)	1 (1–7)	3 (1–5.5)	0.586
Comorbidities			
Hypertension	35 (46.7)	43 (28.9)	0.008 *
Diabetes mellitus	32 (42.7)	49 (32.9)	0.150
Heart failure	6 (8.0)	15 (10.1)	0.616
COPD	2 (2.7)	3 (2.0)	0.775
Liver cirrhosis	6 (8.0)	7 (4.7)	0.319
Hematologic malignancy	5 (6.7)	10 (6.7)	0.990
Solid tumor	11 (4.7)	43 (28.9)	0.019 *
Autoimmune disease	2 (2.7)	4 (2.7)	0.994
Median CCI (IQR)	4 (1–6)	3 (1–6)	0.982
CCI ≥ 3	42 (56)	86 (57.7)	0.806
Potential source of bacteremia			
Pneumonia	41 (54.7)	112 (75.2)	0.002 *
Intra-abdominal	22 (29.3)	70 (47)	0.011 *
Urinary tract	37 (49.3)	72 (48.3)	0.886
Intracranial	27 (36.0)	37 (24.8)	0.081
Skin and soft tissue	9 (12)	25 (16.8)	0.347
Primary bacteremia	4 (5.3)	3 (2.0)	0.178
Hospital-acquired infection	15 (20)	39 (26.2)	0.308
ESBL-producing *E. coli*	41 (54.7)	97 (65.1)	0.130
Previous exposure			
Prior hospitalization	49 (65.3)	107 (71.8)	0.320
Prior ICU stay	10 (13.3)	24 (16.1)	0.585
Prior surgery	22 (29.3)	56 (37.6)	0.221
Prior chemotherapy or radiotherapy	4 (5.3)	17 (11.4)	0.141
Prior corticosteroid use	10 (13.3)	21 (14.1)	0.876
Prior antibiotic use	31 (41.3)	68 (45.6)	0.540
History of hemodialysis	3 (4.0)	12 (8.1)	0.252
Use of invasive procedures or device			
Mechanical ventilation	15 (20)	69 (46.3)	<0.001 *
Central venous catheterization	26 (34.7)	97 (65.1)	<0.001 *
Urinary catheterization	50 (66.7)	131 (87.9)	<0.001 *
Laboratory examination			
Leukocytosis	62 (82.7)	96 (64.4)	0.005 *
Neutropenia	2 (2.7)	13 (8.7)	0.087
Serum albumin < 30 g/L	50 (67.6)	128 (87.1)	0.001 *
Empirical antibiotic treatment	(*n* = 73)	(*n* = 142)	0.626
Cephalosporin	41 (56.2)	76 (53.5)	
Fluoroquinolone	21 (28.8)	45 (31.7)	
BLBLI	11 (15.1)	17 (12)	
Aminoglycoside	0 (0)	2 (1.4)	
Metronidazole	0 (0)	2 (1.4)	
Inappropiate initial antibiotic therapy	36 (50.7)	95 (65.5)	0.036 *
Severity of illness			
Median qSOFA score	1 (0–2)	2 (1–3)	<0.001 *
Median SOFA score (IQR)	4 (2–6)	7 (4.5–8)	<0.001 *
Median PBS (IQR)	0 (0–2)	2 (0–4)	<0.001 *
Vasopressor use	18 (24)	79 (53)	<0.001 *
Median CRP (IQR)	12.89 (5.31–25.32)	20.39 (10.25–28.75)	0.038 *
Median procalcitonin (IQR)	10.8 (2.66–49.8)	24.84 (3.76–62.99)	0.128

Note: * = significant at <0.05. Data are expressed as n (%) unless otherwise stated. IQR: interquartile range; LOS: length of stay; COPD: chronic obstructive pulmonary disease; CCI: Charlson comorbidity index; ESBL: extended-spectrum beta lactamase; ICU: intensive care unit; BLBLI: beta-lactam–beta-lactamase inhibitor; qSOFA: quick sequential organ failure assessment; SOFA: sequential organ failure assessment; CRP: C-reactive protein.

**Table 4:** Risk factors for mortality caused by *E. coli* bacteremia.

Variables	Multivariate Analysis		
	*p*	OR	95% CI for OR
Demographics			
Male gender	0.003	3.646	1.536–8.656
Shorter LOS	<0.001	0.890	0.845–0.936
Comorbidity			
Solid tumor	0.011	3.654	1.346–9.916
Potential source of bacteremia			
Pneumonia	0.015	2.826	1.225–6.520
Use of invasive procedures or devices			
Mechanical ventilation	0.041	2.976	1.045–8.474
Central venous catheterization	0.037	2.498	1.056–5.910
Inappropriate initial antibiotic therapy	0.030	2.403	1.091–5.293
Severity of illness			
SOFA score	<0.001	1.371	1.176–1.598

LOS: length of stay; SOFA: sequential organ failure assessment.

**Table 5:** Bivariate analysis of mortality caused by ESBL *E. coli* bacteremia.

Variables	Bivariate Analysis		
	Survivor (*n* = 41)	Non-Survivor (*n* = 97)	*p*
Demographics			
Male gender	11 (26.8)	49 (50.5)	0.010 *
Median age, years (IQR)	53 (42.5–62)	55 (41.5–63.5)	0.814
Median LOS, days (IQR)	14 (9.5–20)	6 (2–11)	<0.001 *
Comorbidities			
Hypertension	22 (53.7)	27 (27.8)	0.004 *
Diabetes mellitus	19 (46.3)	35 (36.1)	0.259
Heart failure	5 (12.2)	7 (7.2)	0.343
COPD	1 (2.4)	2 (2.1)	0.890
Liver cirrhosis	3 (7.3)	4 (4.1)	0.435
Hematologic malignancy	1 (2.4)	7 (7.2)	0.272
Solid tumor	8 (19.5)	24 (24.7)	0.506
Autoimmune disease	0 (0)	4 (4.1)	0.187
Median Charlson comorbidity index (IQR)	4 (1.5–6)	4 (1–6)	0.622
CCI ≥ 3	24 (58.5)	57 (58.8)	0.980
Potential source of bacteremia			
Pneumonia	24 (58.5)	69 (71.1)	0.149
Intra-abdominal	5 (12.2)	27 (27.8)	0.047 *
Urinary tract	31 (75.6)	62 (63.9)	0.181
Intracranial	1 (2.4)	3 (3.1)	0.834
Skin and soft tissue	9 (22.0)	25 (25.8)	0.634
Mixed infection	16 (39.0)	36 (37.1)	0.832
Primary bloodstream infection	3 (7.3)	2 (2.1)	0.131
Hospital-acquired infection	5 (12.2)	26 (26.8)	0.060
Previous exposure			
Prior hospitalization	29 (70.7)	73 (75.3)	0.580
Prior chemotherapy or radiotherapy	1 (2.4)	8 (8.2)	0.207
Prior corticosteroid use	5 (12.2)	10 (10.3)	0.745
Prior surgery	15 (36.6)	40 (41.2)	0.610
History of hemodialysis	3 (7.3)	9 (9.3)	0.709
Prior antibiotic use	21 (51.2)	55 (56.7)	0.554
Prior ICU stay	5 (12.2)	17 (17.5)	0.434
Use of invasive procedures or devices			
Mechanical ventilation	8 (19.5)	47 (48.5)	0.002 *
Central venous catheterization	31 (75.6)	88 (90.7)	0.019 *
Urinary catheterization	18 (43.9)	68 (70.1)	0.004 *
Laboratory examination			
Leukocytosis	35 (85.4)	70 (72.2)	0.097
Neutropenia	1 (2.4)	5 (5.2)	0.475
Median serum albumin	2.72 (2.47–3.05)	2.33 (2.09–2.65)	<0.001 *
Serum albumin < 30 g/L	28 (70%)	87 (90.6)	0.002 *
Empirical antibiotic treatment			0.708
Cephalosporin	24 (58.5)	56 (59.6)	
Fluoroquinolone	11 (26.8)	24 (25.5)	
BLBLI	6 (14.6)	10 (10.6)	
Aminoglycosides	0 (0)	2 (2.1)	
Metronidazole	0 (0)	2 (2.1)	
Inappropriate initial antibiotic therapy	31 (75.6)	84 (86.6)	0.113
Severity of illness			
Median qSOFA score (IQR)	1 (0–2)	2 (1–3)	<0.001 *
Median SOFA score (IQR)	4 (2–6)	6 (5–8)	<0.001 *
Median PBS (IQR)	0 (0–2)	3 (1–6)	<0.001 *
Vasopressor use	14 (34.1)	53 (54.6)	0.028 *
Median CRP (IQR)	13.11 (8.85–27.66)	20.38 (10.69–32.58)	0.183
Median procalcitonin (IQR)	8.68 (2.89–60.62)	24.60 (3.91–64.03)	0.315

Note: * = significant at <0.05. Data are expressed as n (%) unless otherwise stated. IQR: interquartile range; LOS: length of stay; COPD: chronic obstructive pulmonary disease; CCI: Charlson comorbidity index; ICU: intensive care unit; BLBLI: beta-lactam–beta-lactamase inhibitor; qSOFA: quick sequential organ failure assessment; SOFA: sequential organ failure assessment; PBS: Pitt bacteremia score; CRP: C-reactive protein.

**Table 6:** Risk factors for mortality caused by ESBL *E. coli* bacteremia.

Variables	Multivariate Analysis		
	*p*	OR	95% CI for OR
Demographics			
Male gender	0.007	4.927	1.548–15.683
Shorter LOS	0.004	0.917	0.865–0.972
Comorbidity			
Hypertension	0.003	0.187	0.061–0.569
Use of invasive procedures or device			
Central venous catheterization	0.004	4.885	1.639–14.564
Severity of illness			
SOFA score	<0.001	1.842	1.359–2.496
Vasopressor use, n (%)	0.033	0.227	0.058–0.884

LOS: length of stay; SOFA: sequential organ failure assessment.

## Data Availability

The original data presented in this study are openly available at https://doi.org/10.6084/m9.figshare.29716532.

## Abbreviations

The following abbreviations are used in this manuscript:

AIDS	Acquired immunodeficiency syndrome
AMR	Antimicrobial resistance
BLBLI	Beta-lactam–beta-lactamase inhibitor
BSI	Bloodstream infection
CCI	Charlson comorbidity index
CI	Confidence interval
CLSI	Clinical and Laboratory Standards Institute
COPD	Chronic obstructive pulmonary disease
CRP	C-reactive protein
CVC	Central venous catheter
*E. coli*	*Escherichia coli*
EPV	Events per variable
ESBL	Extended-spectrum beta-lactamase
HIV	Human immunodeficiency virus
ICU	Intensive care unit
IIAT	Inappropriate initial antibiotic therapy
IQR	Interquartile range
LOS	Length of stay
MDRO	Multidrug-resistant organism
OR	Odds ratio
PBS	Pitt bacteremia score
qSOFA	Quick sequential organ failure assessment
SOFA	Sequential organ failure assessment
SPSS	Statistical product and service solutions
UPEC	Uropathogenic *Escherichia coli*
UTI	Urinary tract infection
VIF	Variance inflation factors
WHO	World Health Organization

## References

[1] Husna A., Rahman M.M., Badruzzaman A.T.M., Sikder M.H., Islam M.R., Rahman M.T., Alam J., Ashour H.M. (2023). Extended-Spectrum β-Lactamases (ESBL): Challenges and Opportunities. Biomedicines.

[2] Zhang S., Yang J., Abbas M., Yang Q., Li Q., Liu M., Zhu D., Wang M., Tian B., Cheng A. (2025). Threats across Boundaries: The Spread of ESBL-Positive Enterobacteriaceae Bacteria and Its Challenge to the “One Health” Concept. Front. Microbiol..

[3] Xiao T., Wu Z., Shi Q., Zhang X., Zhou Y., Yu X., Xiao Y. (2019). A Retrospective Analysis of Risk Factors and Outcomes in Patients with Extended-Spectrum Beta-Lactamase-Producing *Escherichia coli* Bloodstream Infections. J. Glob. Antimicrob. Resist..

[4] World Health Organization Global Antimicrobial Resistance and Use Surveillance System (GLASS). https://www.who.int/data/gho/data/themes/topics/global-antimicrobial-resistance-surveillance-system-glass.

[5] Bloodstream Infection Due to *Escherichia coli* Resistant to Third-Generation Cephalosporins, Proportion (%). https://www.who.int/data/gho/data/indicators/indicator-details/GHO/sdg-3.d.2--proportion-of-bloodstream-infections-due-to-selected-antimicrobial-resistant-organisms--median-(-).

[6] Kadariswantiningsih I.N., Rampengan D.D., Ramadhan R.N., Idrisova A., Idrisov B., Empitu M.A. (2025). Antibiotic Resistance in Indonesia: A Systematic Review and Meta-analysis of Extended-spectrum Beta-lactamase-producing Bacteria (2008–2024). Trop. Med. Int. Health.

[7] Limato R., Lazarus G., Dernison P., Mudia M., Alamanda M., Nelwan E.J., Sinto R., Karuniawati A., Rogier van Doorn H., Hamers R.L. (2022). Optimizing Antibiotic Use in Indonesia: A Systematic Review and Evidence Synthesis to Inform Opportunities for Intervention. Lancet Reg. Health Southeast. Asia.

[8] Sianipar O., Asmara W., Dwiprahasto I., Mulyono B. (2019). Mortality Risk of Bloodstream Infection Caused by Either *Escherichia coli* or Klebsiella Pneumoniae Producing Extended-Spectrum β-Lactamase: A Prospective Cohort Study. BMC Res. Notes.

[9] de Maia M.O., da Silveira C.D.G., Gomes M., Fernandes S.E.S., de Santana R.B., de Oliveira D.Q., Amorim F.F.P., de Assis Rocha Neves F., Amorim F.F. (2023). Multidrug-Resistant Bacteria on Critically Ill Patients with Sepsis at Hospital Admission: Risk Factors and Effects on Hospital Mortality. Infect. Drug Resist..

[10] Tsachouridou O., Pilalas D., Nanoudis S., Antoniou A., Bakaimi I., Chrysanthidis T., Markakis K., Kassomenaki A., Mantzana P., Protonotariou E. (2023). Mortality Due to Multidrug-Resistant Gram-Negative Bacteremia in an Endemic Region: No Better Than a Toss of a Coin. Microorganisms.

[11] Namikawa H., Imoto W., Yamada K., Tochino Y., Kaneko Y., Kakeya H., Shuto T. (2023). Predictors of Mortality from Extended-Spectrum Beta-Lactamase-Producing Enterobacteriaceae Bacteremia. Emerg. Microbes Infect..

[12] Ling W., Paterson D.L., Harris P.N.A., Furuya-Kanamori L., Edwards F., Laupland K.B. (2024). Mortality, Hospital Length of Stay, and Recurrent Bloodstream Infections Associated with Extended-Spectrum Beta-Lactamase-Producing *Escherichia coli* in a Low Prevalence Region: A 20-Year Population-Based Large Cohort Study. Int. J. Infect. Dis..

[13] Vance M.K., Cretella D.A., Ward L.M., Vijayvargiya P., Garrigos Z.E., Joyce M., Wingler B. (2023). Risk Factors for Bloodstream Infections Due to ESBL-Producing *Escherichia coli*, Klebsiella spp., and Proteus Mirabilis. Pharmacy.

[14] Baek Y.J., Kim Y.A., Kim D., Shin J.H., Uh Y., Shin K.S., Shin J.H., Jeong S.H., Lee G.W., Lee E.J. (2021). Risk Factors for Extended-Spectrum-β-Lactamase-Producing *Escherichia coli* in Community-Onset Bloodstream Infection: Impact on Long-Term Care Hospitals in Korea. Ann. Lab. Med..

[15] Tsai W.-L., Hung C.-H., Chen H.-A., Wang J.-L., Huang I.-F., Chiou Y.-H., Chen Y.-S., Lee S.S.-J., Hung W.-Y., Cheng M.-F. (2018). Extended-Spectrum β-Lactamase-Producing *Escherichia coli* Bacteremia: Comparison of Pediatric and Adult Populations. J. Microbiol. Immunol. Infect..

[16] (2022). Global Antimicrobial Resistance and Use Surveillance System (GLASS) Report 2022.

[17] Lin T.-C., Hung Y.-P., Lin W.-T., Dai W., Huang Y.-L., Ko W.-C. (2021). Risk Factors and Clinical Impact of Bacteremia Due to Carbapenem-Nonsusceptible Enterobacteriaceae: A Multicenter Study in Southern Taiwan. J. Microbiol. Immunol. Infect..

[18] Altamimi I., Binkhamis K., Alhumimidi A., Alabdulkarim I.M., Almugren A., Alhemsi H., Altamimi A., Almazyed A., Elbih S., Alghunaim R. (2024). Decline in ESBL Production and Carbapenem Resistance in Urinary Tract Infections among Key Bacterial Species during the COVID-19 Pandemic. Antibiotics.

[19] Sharifzadeh Kermani M., Pouradeli S., Sadeghian R., Momen Abadi Z. (2025). Exploring the Impact of Comorbidities and Drug Resistance on Mortality in ICU-Acquired Bloodstream Infections. AMB Express.

[20] Anggraini D., Santosaningsih D., Endraswari P.D., Jasmin N., Siregar F.M., Hadi U., Kuntaman K. (2022). Multicenter Study of the Risk Factors and Outcomes of Bloodstream Infections Caused by Carbapenem-Non-Susceptible Acinetobacter Baumannii in Indonesia. Trop. Med. Infect. Dis..

[21] Hayward M. (2023). The Influence of Host Factors on Susceptibility and Resistance to Infectious Diseases. J. Microbiol. Pathol..

[22] Rockenschaub P., Hayward A., Shallcross L. (2020). Antibiotic Prescribing Before and After the Diagnosis of Comorbidity: A Cohort Study Using Primary Care Electronic Health Records. Clin. Infect. Dis..

[23] Naghavi M., Vollset S.E., Ikuta K.S., Swetschinski L.R., Gray A.P., Wool E.E., Robles Aguilar G., Mestrovic T., Smith G., Han C. (2024). Global Burden of Bacterial Antimicrobial Resistance 1990–2021: A Systematic Analysis with Forecasts to 2050. Lancet.

[24] Alkan S., Balkan I.I., Surme S., Bayramlar O.F., Kaya S.Y., Karaali R., Mete B., Aygun G., Tabak F., Saltoglu N. (2024). Urinary Tract Infections in Older Adults: Associated Factors for Extended-Spectrum Beta-Lactamase Production. Front. Microbiol..

[25] Zhou Y., Zhou Z., Zheng L., Gong Z., Li Y., Jin Y., Huang Y., Chi M. (2023). Urinary Tract Infections Caused by Uropathogenic *Escherichia coli*: Mechanisms of Infection and Treatment Options. Int. J. Mol. Sci..

[26] Al-Groom R. (2025). Incidence of Extended Spectrum Beta-Lactamase (ESBL) Producing *Escherichia coli* Isolated from Women with Urinary Tract Infections in Jordan. Iran. J. Microbiol..

[27] Tanaka M., Hanawa T., Suda T., Tanji Y., Minh L.N., Kondo K., Azam A.H., Kiga K., Yonetani S., Yashiro R. (2025). Comparative Analysis of Virulence-Associated Genes in ESBL-Producing *Escherichia coli* Isolates from Bloodstream and Urinary Tract Infections. Front. Microbiol..

[28] Asmare Z., Awoke T., Genet C., Admas A., Melese A., Mulu W. (2024). Incidence of Catheter-Associated Urinary Tract Infections by Gram-Negative Bacilli and Their ESBL and Carbapenemase Production in Specialized Hospitals of Bahir Dar, Northwest Ethiopia. Antimicrob. Resist. Infect. Control.

[29] Lindblom A., Kiszakiewicz C., Kristiansson E., Yazdanshenas S., Kamenska N., Karami N., Åhrén C. (2022). The Impact of the ST131 Clone on Recurrent ESBL-Producing E. Coli Urinary Tract Infection: A Prospective Comparative Study. Sci. Rep..

[30] Alsehemi A.F., Alharbi E.A., Alammash B.B., Alrais A.I., Elbadawy H.M., Alahmadi Y.M. (2023). Assessment of Risk Factors Associated with Multidrug-Resistant Organism Infections among Patients Admitted in a Tertiary Hospital-a Retrospective Study. Saudi Pharm. J. SPJ.

[31] Gontjes K.J., Gibson K.E., Lansing B.J., Mantey J., Jones K.M., Cassone M., Wang J., Mills J.P., Mody L., Patel P.K. (2022). Association of Exposure to High-Risk Antibiotics in Acute Care Hospitals With Multidrug-Resistant Organism Burden in Nursing Homes. JAMA Netw. Open.

[32] Apisarnthanarak A., Kondo S., Apisarnthanarak P., Mundy L.M. (2020). Risk Factors for Extended-Spectrum Beta-Lactamase–Producing Enterobacteriaceae Enteric Carriage among Abdominal Surgery Patients. Infect. Control Hosp. Epidemiol..

[33] Nasser S., Alnasser Z., Aljuhani O., Alharbi A., Rice J., Alharthi A.F., Kensara R., Al Mutairi F.E., Zaabee D., Alowais S.A. (2025). Exploring Infection Risk Factors and Multi-Drug-Resistant Organisms (MDROs) in Burn Intensive Care Units: A Multi-Centre Case–Control Study. J. Hosp. Infect..

[34] Yassin A., Eid R.A., Mohammad M.F., Elgendy M.O., Mohammed Z., Abdelrahim M.E.A., Abdel Hamied A.M., Binsuwaidan R., Saleh A., Hussein M. (2025). Microbial Multidrug-Resistant Organism (MDRO) Mapping of Intensive Care Unit Infections. Medicina.

[35] Merlinda Veronica R., Kumalawati J., Martin Rumende C., Nainggolan L., Simadibrata M. (2024). Multidrug-Resistant Organisms Infection on Mortality of Burn Patients at Public Hospital X in Jakarta: A Retrospective Study. Kesmas Natl. Public. Health J..

[36] Holmbom M., Möller V., Kristinsdottir L., Nilsson M., Rashid M.-U., Fredrikson M., Berglund B., Östholm Balkhed Å. (2022). Risk Factors and Outcome Due to Extended-Spectrum β-Lactamase-Producing Uropathogenic *Escherichia coli* in Community-Onset Bloodstream Infections: A Ten-Year Cohort Study in Sweden. PLoS ONE.

[37] Septimus E.J., Moody J. (2016). Prevention of Device-Related Healthcare-Associated Infections. F1000Res.

[38] Blomstrom-Lundqvist C., Ostrowska B. (2021). Prevention of Cardiac Implantable Electronic Device Infections: Guidelines and Conventional Prophylaxis. EP Europace.

[39] Battle S.E., Shuping M., Withers S., Justo J.A., Bookstaver P.B., Al-Hasan M.N. (2022). Prediction of Mortality in Staphylococcus Aureus Bloodstream Infection Using Quick Pitt Bacteremia Score. J. Infect..

[40] Al Shaqri E.J., Balkhair A. (2024). Relationship of C-Reactive Protein/Serum Albumin Ratio and QPitt Bacteremia Score with An All-Cause In-Hospital Mortality in Patients with Bloodstream Infections. Cureus.

[41] Al-Hasan M.N., Baddour L.M. (2020). Resilience of the Pitt Bacteremia Score: 3 Decades and Counting. Clin. Infect. Dis..

[42] Zhao S., Wu Y., Dai Z., Chen Y., Zhou X., Zhao J. (2022). Risk Factors for Antibiotic Resistance and Mortality in Patients with Bloodstream Infection of *Escherichia coli*. Eur. J. Clin. Microbiol. Infect. Dis..

[43] Ling W., Furuya-Kanamori L., Ezure Y., Harris P.N.A., Paterson D.L. (2021). Adverse Clinical Outcomes Associated with Infections by Enterobacterales Producing ESBL (ESBL-E): A Systematic Review and Meta-Analysis. JAC Antimicrob. Resist..

[44] Phungoen P., Sarunyaparit J., Apiratwarakul K., Wonglakorn L., Meesing A., Sawanyawisuth K. (2022). The Association of ESBL *Escherichia coli* with Mortality in Patients with *Escherichia coli* Bacteremia at the Emergency Department. Drug Target. Insights.

[45] Pratiwi A.D., Rusli M., Utomo B. (2019). Correlation between ESBL-Producing Bacteria Infection with Sepsis Severity of Patient in Medical Ward of Internal Medicine Department Dr. Soetomo General Hospital in 2016. JUXTA J. Ilm. Mhs. Kedokt. Univ. Airlangga.

[46] Kadri S.S., Lai Y.L., Warner S., Strich J.R., Babiker A., Ricotta E.E., Demirkale C.Y., Dekker J.P., Palmore T.N., Rhee C. (2021). Inappropriate Empirical Antibiotic Therapy for Bloodstream Infections Based on Discordant In-Vitro Susceptibilities: A Retrospective Cohort Analysis of Prevalence, Predictors, and Mortality Risk in US Hospitals. Lancet Infect. Dis..

[47] Aliska G., Nur Utami W. (2024). The Relationship Between Appropriateness of Antibiotic Use Based on the Gyssens Algorithm and Mortality: A Retrospective Cohort Study in Indonesian Tertiary Hospital. Acta Med. Indones-Indones J. Intern Med..

[48] Fazal A.Z., McGovern O.L., Mahon G.W., Lessa F.C., Gler M.T., Garcia J., Festin M.J., Kuntaman K., Parwati I., Siregar C. (2025). Trends in Inpatient Antibiotic Use in Indonesia and the Philippines during the COVID-19 Pandemic. Antimicrob. Steward. Healthc. Epidemiol..

[49] Rahardjoputro R., Amrullah A.W., Rizky W., Ernawati E., Wahyudi A., Widyaningrum N.R. (2025). Rationality Analysis of Antibiotics for Community-Acquired Pneumonia in Adult Inpatients at X Hospital Sukoharjo. Pharmacol. Clin. Pharm. Res..

[50] Savitri A.A., Ni’ma N.S., Susatyo E.B., Nariswara F., Oktaviani S.R. (2024). Rasionalitas Penggunaan Antibiotik Empiris Pada Pasien Pneumonia Di Bangsal Rawat Inap Rumah Sakit Bhakti Wira Tamtama Semarang. Media Farm. Indones..

[51] Fath S., Fath Thoriq S., Syafhan N.F., Luthfi Aziz M. (2024). The Impact of Appropriateness of Empirical Antibiotic in Hospitalized Pneumonia Patients on Clinical Outcomes and Length of Stay. J. Univers. Stud..

[52] Allel K., Peters A., Furuya-Kanamori L., Spencer-Sandino M., Pitchforth E., Yakob L., Munita J.M., Undurraga E.A. (2024). Impact of Inappropriate Empirical Antibiotic Therapy on In-Hospital Mortality: A Retrospective Multicentre Cohort Study of Patients with Bloodstream Infections in Chile, 2018–2022. BMJ Public Health.

[53] Handal N., Whitworth J., Nakrem Lyngbakken M., Berdal J.E., Dalgard O., Bakken Jørgensen S. (2024). Mortality and Length of Hospital Stay after Bloodstream Infections Caused by ESBL-Producing Compared to Non-ESBL-Producing E. Coli. Infect. Dis..

[54] Tomidokoro D., Asai Y., Hayakawa K., Kutsuna S., Terada M., Sugiura W., Ohmagari N., Hiroi Y. (2023). Comparison of the Clinical Characteristics and Outcomes of Japanese Patients with COVID-19 Treated in Primary, Secondary, and Tertiary Care Facilities. J. Infect. Chemother..

[55] Ternavasio-De La Vega H.G., Castaño-Romero F., Ragozzino S., Sánchez González R., Vaquero-Herrero M.P., Siller-Ruiz M., Spalter-Glicberg G., Ramírez-Baum C., Rodríguez-Rodríguez S., García-Sánchez J.E. (2018). The Updated Charlson Comorbidity Index Is a Useful Predictor of Mortality in Patients with Staphylococcus Aureus Bacteraemia. Epidemiol. Infect..

[56] (2022). Performance Standards for Antimicrobial Susceptibility Testing.

[57] Sanguinetti M., Posteraro B., Spanu T., Ciccaglione D., Romano L., Fiori B., Nicoletti G., Zanetti S., Fadda G. (2003). Characterization of Clinical Isolates of *Enterobacteriaceae* from Italy by the BD Phoenix Extended-Spectrum β-Lactamase Detection Method. J. Clin. Microbiol..

